# *Taenia* sp. in human burial from Kan River, East Siberia

**DOI:** 10.1590/0074-02760160442

**Published:** 2017-03-27

**Authors:** Sergey Mikhailovich Slepchenko, Sergey Nikolaevich Ivanov, Anton Vasilevich Vybornov, Tsybankov Alexander Alekseevich, Slavinsky Vyacheslav Sergeyevich, Danil Nikolaevich Lysenko, Vyacheslav Evgenievich Matveev

**Affiliations:** 1 Ltd PaleopoiskNovosibirskRussiaLtd Paleopoisk, Novosibirsk, Russia; 2 Institute of the Problems of Northern DevelopmentTyumenRussiaInstitute of the Problems of Northern Development, Siberian Branch of the Russian Academy of Sciences, Tyumen, Russia; 3 Institute of Archaeology and EthnographyNovosibirskRussiaInstitute of Archaeology and Ethnography, Siberian Branch of the Russian Academy of Sciences, Novosibirsk, Russia; 4 Novosibirsk State UniversityNovosibirskRussiaNovosibirsk State University, Novosibirsk, Russia; 5 Ltd Krasnoyarsk’s GeoarcheologyKrasnoyarskRussiaLtd Krasnoyarsk’s Geoarcheology, Krasnoyarsk, Russia

**Keywords:** *Taenia* sp, archaeoparasitology, paleoparasitology, East Siberia, Bronze Age

## Abstract

We present an arhaeoparasitological analysis of a unique burial from the Neftprovod II burial ground in East Siberia, which dated from the Bronze Age. Analysis of a sediment sample from the sacral region of the pelvis revealed the presence of *Taenia* sp. eggs. Because uncooked animal tissue is the primary source of *Taenia*, this indicated that the individual was likely consuming raw or undercooked meat of roe deer, red deer, or elk infected with *Taenia*. This finding represents the oldest case of a human infected with *Taenia* sp*.* from Eastern Siberia and Russia.

Nefteprovod I and the adjacent Nefteprovod II burial grounds were excavated as part of a salvage project at the Anzhevsk archaeological site. This site is located near the former Anzhevka settlement on the right bank of the Kan River southeast of Kansk in the Krasnoyarsk region (56º11’40.20”N. 95º49’11.94”W) (Fig. 1). Fifteen burials were recovered during the July and August 2015 excavation season. The majority of the burials were covered by a layer of stones. Bodies were typically buried in a supine position with the heads oriented eastward towards the Kan River. Several burials contained ornaments, as well as metal, bone, and stone tools. Although the recovered artefact styles have no direct analogies, similar bronze and bone artefacts and ceramic vessels are found along the middle Yenisei River and date to the Late Bronze Age. Based on these artefact similarities and the burial style, the Nefteprovod I and II burial grounds likely belong to the Karasuk culture, which succeeded the Andronovo culture in this region during the later portion of the second millennium BC. The Karasuk culture lasted from approximately 1500 to 800 BC. Burial #13 from the Nefteprovod II archeological site differed considerably from the other burials in its funerary style. The body was tightly flexed inside a tight burial pit with uneven margins. Grave goods were absent. The stonework overlaying the burial dated back to the Late Bronze Age, indicating that the burial came from earlier cultural strata, likely the middle Bronze Age (2000 to 1000 BC) (Fig. 1). The positioning of the body in a foetal position is unique in the region of the Kansk forest steppe. Similar burials under stonework have been found in adjacent territories of the Baikal and Angara regions, and they are associated with the Glazkovskaya culture, which dated from 2000 to 1300 BC. (Okladnikov 1975a, 1975b, Dudarёk & Lohov 2014, Derevianko et al. 2015). Tightly flexed burials were also part of the Andronovskaya culture from the Minusinsk Basin (Maksimenkov 1978).

**Fig. 1:**
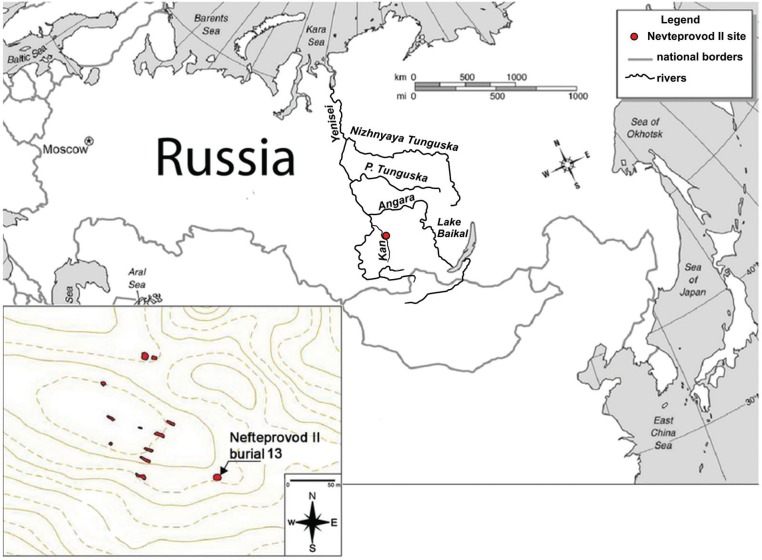
: location of the archaeological site Neftprovod II. Distribution of burials on the archaeological site Neftprovod II in 2015. Burials are marked in red.

The skeleton from burial #13, which provided the material for this analysis, was discovered inside excavation sector 147. The burial had an uneven shape, and measured 1.6 m by 2.0 m in size and 0.4 m in depth (Fig. 2). We found an oval-shaped stonework above the burial, measuring 1.6 m by 2.0 m with a thickness of 15-25 cm. This stonework overlaying the burial was constructed from massive unmodified stones. Numerous artefact fragments, including those of pots, ornaments, and bone implements, have been recovered from the spaces between the stone. The burial was discovered at a depth of about 0.1-0.15 m underneath the stonework. The body was oriented along a southeast-northwest axis parallel to the river. The head pointed towards the northwest, and the face was positioned to look directly at the river. The skeleton was placed on its right side with the legs bent at the knees and pressed against the stomach. The arms were bent at the elbows with the hands positioned underneath the right side of the mandible.

**Fig. 2:**
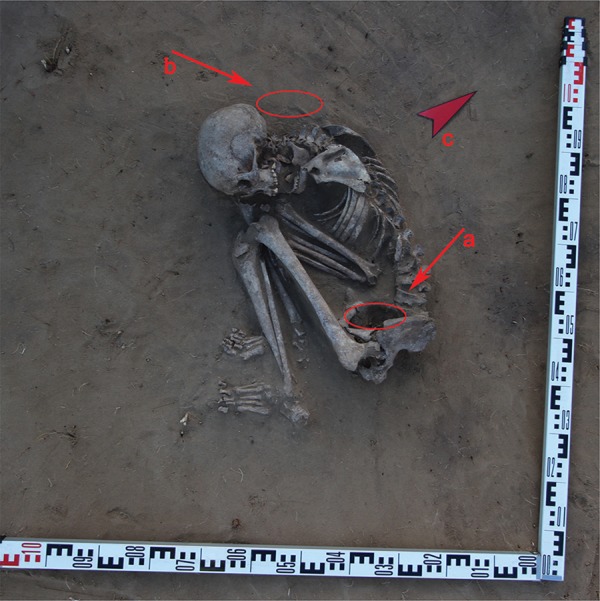
: skeleton from burial #13 at Neftprovod II was excavated and sampled. (a) The location where the sediment sample was collected for the paleoparasitological analysis; (b) the location where the control soil sample was collected; (c) the arrow indicates the direction of north.

A sediment sample was collected from inside the pelvic region and sacral foramina, which served as the material for this analysis. The sample was collected from the remains of the human body from inside the sacral foramina according to standard procedures. Briefly, all superficial and surrounding sediments were carefully removed layer by layer to avoid contamination, making the presence of non-zoonotic *Taenia* highly unlikely. The average weight of the collected sediment samples was 150 g. All samples were vacuum-packed in the field and transported to the research facility at the Institute of the Problems of Northern Development, Siberian Branch of the Russian Academy of Sciences for further analysis. To control for environmental contamination, we collected additional sediment samples from the area around the skull, as well as from inside the burial pit 1 m away from the body (Fig. 2).

The samples were processed in the laboratory following standard protocols (Callen & Cameron 1960, Araújo et al. 1998). Briefly, 50 g of dry sediment from each sample was placed into an 800 mL Bunsen beaker containing a solution of 0.5% sodium phosphate (Na_3_PO_4_). The supernatant was elutriated three times over the course of a week. This was followed by sifting of the residue using a 200-µM sieve. Sample separation was performed in centrifugal tubes. The residue was gathered by repeated centrifugation for seven minutes at 1500 revolutions per min. Since the residue contained a significant amount of fine-grained sand, we used hydrofluoric acid to dissolve it. Following this treatment, each sample was centrifuged, the hydrofluoric acid (49%) was removed, and the organic fraction was detached. After that, we added glycerin until the whole residue was covered. We stirred the residue carefully and warmed it inside the tube using a water bath set at 80ºC for 10 min. The hot tubes were centrifuged for seven min at 1500 revolutions per min. Afterwards, glycerin and the remaining water were elutriated, as described by Dufour and Le Bailly (2013).

The small amount of organic residue allowed us to prepare 19 microslides from the samples we obtained. Microscopic examination was conducted using the AxioSkop 40 and MicMed 2 var. 2 microscopes under 80x and 400x magnifications. AxioVision 4.6 and Scope Photo 3.0 were used for measuring the observed features.

Microscopic examination of the microslides revealed three helminth eggs. These eggs were a light brown colour, spherical in shape, and displayed a thick, radially striated eggshell. The egg dimensions averaged 35.3 μM by 30.7 μM. These morphological characters suggested that the eggs belong to the genus *Taenia* spp. (Fig. 3) (Ash & Orihel 2007). Control samples, which we collected at the skull area in the burial, were all negative for eggs with this morphology.

**Fig. 3:**
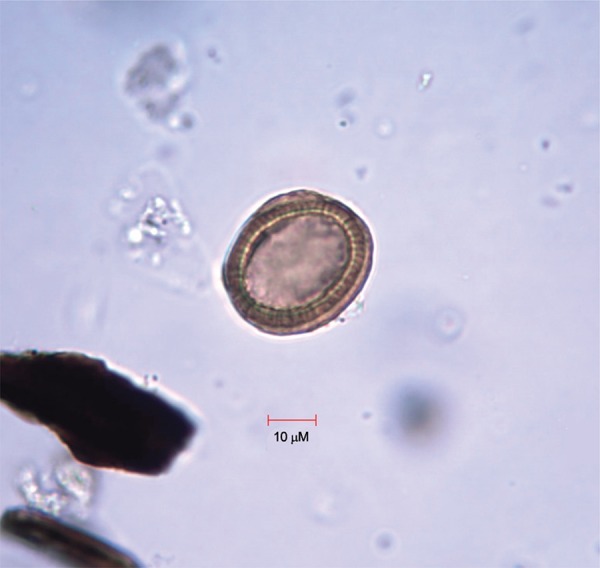
: eggs morphologically similar to *Taenia* sp. found in the remains from burial #13 at the Neftprovod II burial ground of Bronze Age East Siberia.

Paleoparasitological data show that *Taenia* spp*.* has been geographically and chronologically widespread. *Taenia* spp*.* eggs have been found in mummies, coprolites, and in the pelvic regions of individuals from several chronologically different archaeological sites in Europe (Switzerland, Austria, France, and Cyprus), Asia (Japan and eastern Russia), Africa (Egypt), as well as from sites in South and North America (Reyman et al. 1977, Dommelier et al. 1998, Gonçalves et al. 2003, Le Bailly 2005, Sianto et al. 2009).

Paleoparasitological data from Russia are scarce and, thus far, limited to the discovery of *Taenia saginata* eggs in an individual from the Vesakoyaha III burial ground affiliated with the Nenet indigenous ethnic group of northwestern Siberia, which dates back to the 19th century (Slepchenko et al. 2016). In that study, the analysis suggested that the main source of the tapeworm infection was through the consumption of raw reindeer brain.

Humans can be infected by three species of *Taenia*. *T. saginata* is one of these species, and it has intermediary hosts that include cattle and wild cloven-footed ruminants. *T. solium* and *T. asiatica* also infect humans, and their intermediary hosts are the domestic pig and wild boar. The eggs of these parasites are morphologically alike (Ash & Orihel 2007). The infection is caused by the consumption of eggs from contaminated, undercooked, or uncooked beef and pork, as well as eating the meat of wild animals.

Because domesticated cattle and pigs serve as the primary source of *Taenia* infections in contemporary human populations, and because of the absence of domesticated animals in the archaeological record of the site, it seems likely that the *Taenia* infection that we documented was contracted through the consumption of raw or poorly cooked tissues from a wild animal, likely an ungulate. Elk (*Alces alces*), for instance, have been documented to carry *Taenia* (Shestakov & Novikov 2011).

Bones of artiodactyls, including elk, roe deer (*Capreolus capreolus*), and red deer (*Cervus elaphus*), have been recovered from archaeological sites in the Kan River Basin and adjacent areas in Eastern Siberia (Yermolov 1978, Khamzina 1979, Bocharov et al. 2014, Timoshenko 2014). The habitat of elk is very broad and covers all forest and forest steppe zones in Western and Eastern Siberia (Geptner et al. 1961). According to paleozoological investigations of archeological sites close to the Nefteprovod II burial ground, only a few elk bones were found. However, bones of roe deer and red deer were abundant (Khamzina 1979).

Roe deer and red deer represent two widespread cloven-hoofed artiodactyls in Western and Eastern Siberia. Fluctuations in the numbers of these animals have been reported, corresponding to the changes in their habitat in the area under study (Geptner et al. 1961). Presently, roe deer and red deer are widespread in the area of the Nefteprovod II archaeological site. According to paleozoological data, these deer were some of the most prevalent animals in this area during the Mesolithic to the Late Middle Ages, and they were probably hunted (Yermolov 1978, Khamzina 1979).

The habitat of wild boar, which could also serve as a zoonotic source of *Taenia* helminth infections in humans, is found more than 150 km south of the archaeological site. It extends toward the Kungus River, which forms the right tributary of the Kan River (Geptner et al. 1961). Wild boar are not known to have adapted to the harsh snowy winters of our study region (Formozov 2010). Moreover, descriptions of wild boar habitats in the Kansk-Rybinsk Basin in ancient times are scarce. Paleozoological studies indicate that the bones of wild boar are extremely rare among the osteological materials found in chronologically similar archaeological sites from this region (Khamzina 1979). Nonetheless, a single finding of wild boar bones during the Neolithic (Yermolov 1978) suggests that these animals may have ventured into our area of the study during this era.

Based upon the available zooarchaeological data, roe deer appear to be the main animals hunted during the Bronze Age. Bones of roe deer, red deer, and elk account for 50% to 95% of all animal bones recovered from Bronze Age archaeological sites in this region (Yermolov 1978, Khamzina 1979). Although the aforementioned animals can serve as intermediate hosts for *T. saginata* (Shestakov & Novikov 2011), we cannot exclude the possibility that the individual from burial site #13 was infected by another species of *Taenia*. Since the bones of wild boar constituted less than 1% of the total animal bones recovered from archaeological sites in this region (Yermolov 1978, Khamzina 1979), it seems very unlikely that the recovered helminth eggs belonged to a species of *Taenia* for which suidae serve as intermediate hosts. This would include *T. solium* and *T. asiatica*.

To summarise, we report the earliest direct evidence of an endoparasitic infection in a human living in Eastern Siberia during the early/middle Bronze Age. Paleoparasitological analysis revealed eggs of *Taenia* sp. in the sediment sample taken from the pelvic region of the individual from burial #13 in the Nefteprovod II burial ground. This discovery of *Taenia sp.* is the earliest in Siberia. The individual was most likely infected with the tapeworm through the consumption of raw brain tissue from a wild angulate that was infected with *Taenia* sp. This angulate was possibly roe deer, red deer, or elk.

## References

[B1] Araújo A, Reinhard K, Bastos OM, Costa LC, Pirmez C, Iñiguez AM, et al. Paleoparasitology: perspectives with new techniques. Rev Inst Med Trop São Paulo. 1998; 40(6): 371-6. 10.1590/s0036-46651998000600006 10436657

[B2] Ash LR, Orihel TC. Atlas of human parasitology. 5th ed. Chicago: American Society for Clinical Pathology Press; 2007. 540 pp.

[B3] Bocharov EH, Timoshenko AA, Saveliev NA. The transition from the Mesolithic to the Neolithic: the chronological limits and the degree of cultural continuity (based on the multi-layer location Kazachka I). Bull. Novosibirsk State University. Series: History, Philology. 2014; 13(5): 125-34. (in Russian).

[B4] Callen EO, Cameron TWM. A prehistoric diet revealed in coprolites. New Scientist. 1960; 8(190): 35-40.

[B5] Derevianko AP, Tsybankov AA, Postnov AB, Slavinskiy VS, Vibornov AB, Zolnikov ID, et al. Boguchanskaya archaeological expedition: field research (2007-2012). Novosibirsk: Publisher IAE SB RAS; 2015. 564 pp. (in Russian).

[B6] Dommelier S, Bentrad S, Bouchet F, Paicheler JC, Pétrequin P. Parasitoses liées à l’alimentation chez les populations du site néolithique de Chalain (Jura, France). Anthropozool. 1998; 27: 41-9.

[B7] Dudarёk JV, Lohov NA. The burial complexes of the Bronze Age Northern Angara region. Questions chronology and cultural affiliation. News of Irkutsk State University. 2014; 7: 54-80.

[B8] Dufour B, Le Bailly M. Testing new parasite egg extraction methods in paleoparasitology and an attempt at quantification. Int J Paleopathol. 2013; 3(3): 199-203. 10.1016/j.ijpp.2013.03.008 29539456

[B9] Formozov AN. Snow cover in the life of mammals and birds. Moscow: Publisher Moscow Society of Naturalists; 2010. 288 pp. (in Russian).

[B10] Geptner VG, Bannikov AG, Hoffmann RS, Nasimovich AA. Mammals of the Soviet Union. Vol. I. Moscow: Higher School; 1961. 1000 pp. (in Russian).

[B11] Gonçalves MLC, Araújo A, Ferreira LF. Human intestinal parasites in the past: new findings and a Review. Mem Inst Oswaldo Cruz. 2003; 98(Suppl. 1): 103-18. 10.1590/s0074-02762003000900016 12687769

[B12] Khamzina A. Results of the analysis of osteological collection multilayer site Kazachka (Krasnoyarsk region). In: Abstracts reporting scientific-theoretical conference. Irkutsk: University Press; 1979. 48-9.

[B13] Le Bailly M. Evolution de la relation hôte/parasite dans les systémes lacustres nord alpins au Néolithique (3900-2900 BC), et nouvelles données dans la délection des paléoantigénes de Protozoa [PhD Thesis]. Université de Reims Champagne-Ardenne Reims; 2005. 276 pp.

[B14] Maksimenkov GA. Andronovo culture on the Yenisei. Leningrad: Nauka; 1978. 191 pp. (in Russian).

[B15] Okladnikov AP. Neolithic sites of Central Angara (from Serovo to Bratsk). Novosibirsk: Nauka; 1975a. 328 pp. (in Russian).

[B16] Okladnikov AP. Neolithic sites of Central Angara (from the mouth of the White River to Ust-Uda). Novosibirsk: Nauka; 1975b. 320 pp. (in Russian).

[B17] Reyman TA, Zimmerman MR, Lewin PK. Autopsy of an Egyptian mummy (Nakht-ROM I). Histopathologic investigation. CMAJ. 1977; 117: 461-76.

[B18] Shestakov SV, Novikov TV. Description of parasitogenic bubbles, exposed at veterinary and sanitary examination of carcasses of Elk. Bull. Buryat State Agricultural Academy. 2011; 1: 29-34. (in Russian).

[B19] Sianto L, Chame M, Silva CS, Gonçalves ML, Reinhard K, Fugassa M, et al. Animal helminths in human archaeological remains: a review of zoonoses in the past. Rev Inst Med Trop São Paulo. 2009; 51(3): 119-30. 10.1590/s0036-46652009000300001 19551285

[B20] Slepchenko SM, Ivanov SN, Bagashev AN, Tsybankov AA, Slavinsky VS. Traditional living habits of the Taz Tundra population: a paleoparasitological study. Korean J Parasitol. 2016; 54(5): 617-23. 10.3347/kjp.2016.54.5.617 PMC5127544 27853118

[B21] Timoshenko AA. Chronology and periodization of the Stone Age and Kansk- Rybinsk Basin. News of Irkutsk State University. Series Geoarchaeology, Ethnology and Anthropology. 2014; 10: 27-49 (in Russian).

[B22] Yermolov HM. Theriofauna Angara valley in the late Anthropogen. Novosibirsk: Nauka; 1978. 220 pp. (in Russian).

